# Association of Intrarenal B-Cell Infiltrates with Clinical Outcome in Lupus Nephritis: A Study of 192 Cases

**DOI:** 10.1155/2012/967584

**Published:** 2012-06-25

**Authors:** Yan Shen, Chuan-Yin Sun, Feng-Xia Wu, Yi Chen, Min Dai, Yu-Cheng Yan, Cheng-De Yang

**Affiliations:** ^1^Department of Rheumatology, Renji Hospital, Shanghai Jiaotong University School of Medicine, 145 Shan Dong Zhong Road, Shanghai 200001, China; ^2^Department of Nephrology, Renji Hospital, Shanghai Jiaotong University School of Medicine, 145 Shan Dong Zhong Road, Shanghai 200001, China

## Abstract

*Background*. Lupus nephritis (LN) remains a major cause of morbidity and end-stage renal disease. Dysfunction of B lymphocytes is thought to be important in the pathogenesis of SLE/LN. Intrarenal B cells have been found in several forms of inflammatory kidney diseases although their role in LN renal is not well defined. *Methods*. Intrarenal B cells were analyzed in 192 renal biopsies from patients diagnosed with lupus nephritis. Immunohistochemical staining of serial sections was performed for each LN patient using CD20, CD3, and CD21 antibodies. *Results*. Intrarenal B cells were more likely to be associated with class IV LN and were mainly distributed in the renal interstitium, with very few in the glomerulus. The systemic lupus erythematosus disease activity index (SLEDAI), blood urea nitrogen, and serum creatinine levels were all significantly greater in the LN-B cell groups (all *P* < 0.05). LN renal activity and chronicity indices correlated with B-cells infiltrates (all *P* < 0.0001). Renal biopsies were classified into four distinct categories according to the organizational grade of inflammatory cell infiltrates. Germinal center- (GC-) like structures were not identified in any LN biopsies. *Conclusion*. It is hypothesized that intrarenal B cells enhance immunological responses and exaggerate the local immune response to persisting autoimmune damage in the tubulointerstitium.

## 1. Background

Lupus nephritis (LN) is the main cause of morbidity and mortality in systemic lupus erythematosus (SLE) [1]. LN develops in up to 60% of SLE patients during the course of the disease and its treatment remains a challenge [2]. LN is characterized by immune complex deposition and inflammation in glomeruli and the tubulointerstitium. Many studies have indicated that systemic loss of B-cell tolerance results in the local deposition of immune complexes [3, 4].

Intrarenal mononuclear cells have long been believed to be composed mainly of monocytes and T cells. Classically, B cells have been considered to exert long-range effects mostly via activation in secondary lymphoid organs such as lymph nodes and spleen, with subsequent proliferation and differentiation into antibody-producing plasma cells. Consequently, few researchers have focused on the role of B cells as part of the renal infiltrate. However, a high prevalence of intrarenal B cells has been noted in immune-mediated diseases, such as renal transplant rejection and glomerulonephritis [5–7] thus indicating that local B-cell infiltrates play a role in tissue injury such as tissue fibrosis, neolymphangiogenesis, and ectopic lymphogenesis [8]. Investigations in the MRL/*lpr* mouse model of lupus nephritis have indicated that B cells exert a pathogenic role in the absence of soluble autoantibody production [9]. Moreover, Steinmetz et al. observed that the majority of B cells in lupus nephritis patients displayed a mature non-antibody-producing phenotype with antigen-presenting ability [10]. Recently, the contribution of B cells to the formation of lymphoid-like structures in renal tissue has been proposed [10, 11]. B cells within these lymphoid structures secrete autoantibodies [12] and are required locally to maintain activated T cells [11]. These observations provide evidence of the functional importance of this intrarenal lymphoid tissue, although the clinical impact remains to be elucidated. It is hypothesized that intrarenal B cells form part of a local system with pivotal involvement in the pathogenesis of lupus nephritis. No detailed data are currently available. In this study, renal B-cell infiltrates were analyzed in a large number of human lupus nephritis patients to reveal the relationship between B-cell infiltration and clinical parameters in order to further elucidate the mechanism in LN. 

## 2. Methods

### 2.1. Patients

A prospective study of 192 patients who attended the Department of Rheumatology of Renji Hospital at the Shanghai Jiaotong University School of Medicine was carried out. All patients fulfilled the American College of Rheumatology classification criteria for the diagnosis of SLE [13]. Clinical evidence of LN was obtained in all cases and LN diagnosis was confirmed by examination of renal biopsy specimens. Plasma samples were collected on the day of renal biopsy. The following demographic, clinical, and serologic data were collected at the time of renal biopsy: sex, age, duration of SLE and LN, systemic lupus erythematosus disease activity index (SLEDAI), 24 h proteinuria, levels of blood urea nitrogen, serum creatinine, serum C3, C4. The presence or absence of antinuclear antibodies (ANA), antiSm, anti-ribonucleoprotein (anti-RNP), anti-double-stranded DNA (anti-dsDNA), and antinucleosome antibodies was determined. SLEDAI was used to estimate global disease activity.

Informed patient consent was obtained prior to participation in the study, and the study protocol was approved by the institutional review board of Shanghai Jiaotong University.

### 2.2. Histology of Renal Biopsy Samples 

All patients underwent ultrasound-guided renal needle biopsy. Renal tissues obtained by biopsy were fixed in 10% neutral-buffered formalin, dehydrated gradually, and embedded in paraffin. Paraffin-embedded tissue sections were stained with hematoxylin and eosin, periodic acid-Schiff, Masson's trichrome, and periodic acid-silver methenamine. Small portions of fresh renal tissue were snap-frozen, and 4 *μ*m cryostat-cut sections were incubated with fluorescein isothiocyanate (FITC-) conjugated rabbit antisera against human IgG, IgA, IgM, C1q, and C3 (Dako, Denmark), and the direct immunofluorescence of these sections was examined. The biopsy specimens were classified using the International Society of Nephrology/Renal Pathology Society (ISN/RPS) 2003 classification of LN [14]. 

### 2.3. Activity and Chronicity Indices of Renal Tissue Injury

Renal tissue injury was evaluated on the basis of activity and chronicity indices according to methods reported by Austin et al. [15]. Activity index was calculated as the sum of the scores (on a scale of 1–3) of endocapillary proliferation, karyorrhexis, fibrinoid necrosis (the score was multiplied by 2), cellular crescents (the score was multiplied by 2), hyaline deposits, leukocyte exudation, and interstitial inflammation. The score of the chronicity index was the sum of the scores (on a scale of 1–3) for glomerular sclerosis, fibrous crescents, tubular atrophy, and interstitial fibrosis.

### 2.4. Immunohistochemical Staining of Renal Biopsy Samples

Immunohistochemical staining of serial sections was performed for each LN patient using the following antibodies: CD20 (L26, Dako; Glostrup, Denmark), CD3 (Dako; Glostrup, Denmark), and CD21 (1F8; Dako; Glostrup, Denmark). 

Paraffin-embedded tissue sections were placed on positively charged slides and incubated in a stove at 60°C for 1 h. Sections were deparaffinized and rehydrated through a series of washes with xylene and graded alcohols. Endogenous peroxidase was blocked by treatment with 3% H_2_O_2_ for 30 min. Antigen retrieval was performed by flooding the slides with 10 mM citrate buffer (pH 6.0) and heating in a microwave at 1,000 W for 10 min. Primary mouse monoclonal anti-human CD20, CD3, and CD21 antibodies were applied to the slides at a dilution of 1 : 50 in 1% bovine serum albumin/phosphate buffered saline (BSA/PBS) and subsequently incubated overnight at room temperature. Slides were then incubated with a secondary goat anti-mouse IgG antibody (H+L; Dako) for 30 min. Sections were washed with PBS (pH 7.4) between each step (3 times for 5 min). Finally, sections were counterstained with Mayer's hematoxylin, air-dried, cleared in xylene, and coverslipped. 

### 2.5. Quantification of Immunofluorescence and Immunohistochemical Staining Scores 

The intensity of glomerular staining of IgG, IgA, IgM, C3, and C1q was semi-quantitatively assigned a score of 0, 1, or 2. 

**Figure 1:**
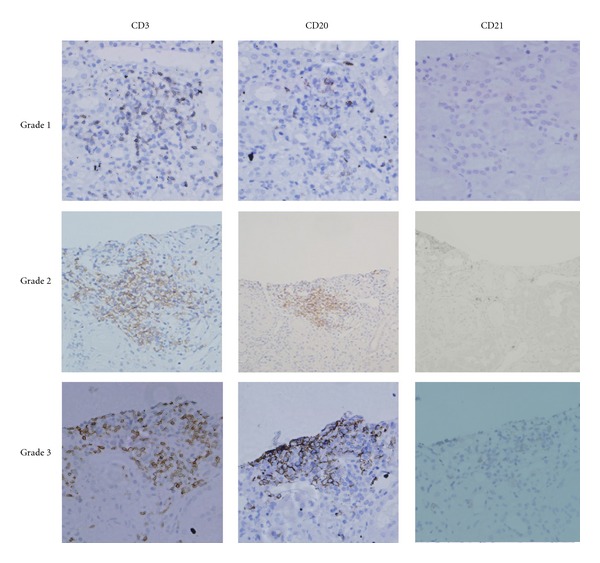
Microanatomical organization of inflammatory infiltrates. Serial staining for CD3, CD20, and CD21 allowed classification into four different grades. Grade 0 aggregates consist of T cell infiltrates alone (no B cells) (not shown). Grade 1 aggregates consist of scattered T- and B-cell infiltrates (original magnification: ×400). Grade 2 aggregates show a cluster-like structure. No T- and B-cell zones are evident. Grade 3 aggregates show clearly distinguishable T- and B-cell areas (original magnification: ×200).

Results of 192 LN patient renal biopsies were classified according to the organizational stage of inflammatory cell infiltrates as described by Steinmetz et al. [10]. A scattered pattern of intrarenal CD20-positive B cells were graded as 1. Nodular aggregates consisting of CD3-positive T cells and CD20-positive B cells without microanatomical compartmentalization were graded as 2. Distinct T-cell and B-cell zones without a central dendritic cell network of aggregates were graded as 3. Aggregates with the highest level of microanatomical organization, consisting of distinct T- and B-cell compartments with a central network of CD21-positive follicular dendritic cells (fDCs) were graded as 4. Typical examples of these grades of infiltrates are shown in Figure 1. All biopsy specimens were scored by a renal pathologist with no prior knowledge of the clinical and laboratory analysis details of patients. 

### 2.6. Statistical Analysis

All statistical analyses were performed using SPSS version 11.0 software (SPSS Inc., Chicago, USA). Categorical variables were compared using Fisher's exact test or chi-square test. Differences between the median values of defined patient groups were compared using the nonparametric Mann-Whitney *U* test. A Spearman's rank correlation was used to detect correlations among different study parameters. *P* < 0.05 was considered statistically significant.

## 3. Results

### 3.1. Demographic, Clinical Characteristics, and Laboratory Results of LN Patients

Firstly, in this prospective study, 192 LN patients (167 women and 25 men; mean age ± SD: 33 ± 13 years) were separated into 2 groups: LN with intrarenal B cells (LN-B group) and LN without intrarenal B cells (LN-non-B group). The LN-B group comprised 118 (61.5%) patients (101 women and 17 men, 34 ± 14 years) and the LN-non-B group comprised 74 (38.5%) patients (66 women and 8 men, 33 ± 14 years). No significant difference was detected between the two groups in terms of age or gender (*P* > 0.05). SLEDAI, blood urea nitrogen, and serum creatinine levels were all significantly greater in the LN-B-cell group than in the LN-non-B-cell group (all *P* < 0.05). However, the duration of SLE or LN, the level of C3/C4, and the proteinuria (g/24 h), were not statistically different between the two groups. Furthermore, no association between intrarenal B-cell infiltration and anti-dsDNA antibodies was identified (all *P* > 0.05; Table 1). No association between intrarenal B-cell infiltration and the level of ANA, anti-Sm, or antinucleosome antibodies between the two groups was identified (all *P* > 0.05; data not shown). 

### 3.2. Microanatomical Organization of Inflammatory Infiltrates

All biopsy samples from LN patients were stained for CD20 as a pan-B-cell marker, in addition to CD3 and CD21 as T cell and fDC markers, respectively. Serial sections were prepared and examined for each patient as previously described [10]. Infiltrates consisting of T cells alone (no B cells, the same to non-B-cell group) were graded as 0. Scattered B- and T-cells were graded as 1. Nodular aggregates without distinct T- and B-cell zones were graded as 2. Leukocyte clusters in which distinct T- and B-cell regions had formed were graded as 3. The highest organizational level clearly showed separate B- and T-cell compartments with a central fDC network that is a classical feature of germinal centers of lymph follicles in secondary lymphoid tissue. These aggregates were graded as 4. B cells were mainly distributed in renal tubules, renal interstitial vessels and interstitial distribution, with very few in the glomeruli (Figure 1). No grade 4 infiltration was observed in biopsies from lupus nephritis patients in this study. 

### 3.3. Inflammatory Infiltrates and Renal Histology

The distribution of the ISN/RPS classification of the 192 patients was as follows: seven were class I, five were class II, 13 were class III, 71 were class IV, 43 were class V, 28 were class (III + V), and 24 were class (IV + V). The distribution of the LN-B-cell group was as follows:, one of the biopsies was class I, one was class II, five were class III, 56 were class IV, 20 were class V, 15 were class III + V, and 19 were class IV + V. When compared with LN-non-B-cell group, the LN-B-cell group was more likely to be associated with class IV LN (*P* < 0.05). The activity and chronicity indices were also significantly higher in the LN-B-cell group than in the LN-non-B-cell group (all *P* < 0.001), with median (25–75th percentile) activity index values of 5 (3–7) and 2 (1–4.5), respectively, and median (25–75th percentile) chronicity index values of 3 (1–4) and 1 (0–2.5), respectively (Table 2).

### 3.4. Clinical Features of Different Intrarenal Cell Grades

**Figure 2:**
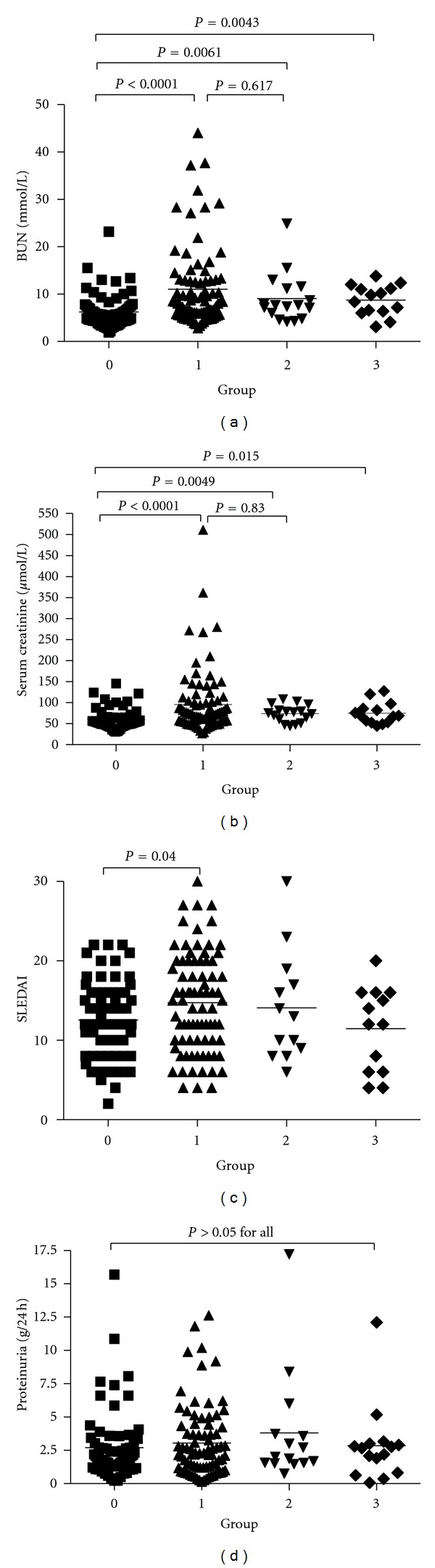
Serum blood urea nitrogen and creatinine levels, SLEDAI, and 24-hour urinary protein level in four different groups of LN patients.

Examination of renal biopsy samples from 192 patients with LN revealed. There were 74 (38.5%) patients in grade 0, 86 (44.8%) patients in grade 1, 17 (8.9%) patients in grade 2, and 15 (7.8%) patients in grade 3. No patients were identified in grade 4. The clinical parameters for blood urea nitrogen levels for patients in grade 0 was lower than those in other grades (all *P* < 0.05). There were no statistical differences between grades 1, 2 and 3 (all *P* > 0.05) (Figure 2(a)). An identical pattern was observed in serum creatinine levels (Figure 2(b)). In the LN-B-cell grade 1 group, the SLEDAI level was higher than the grade 0 group (*P* < 0.05) although there were no statistical differences between the other groups (all *P* > 0.05) (Figure 2(c)). No statistical differences were identified between any groups in the 24-hour urinary protein levels (all *P* > 0.05) (Figure 2(d)) or in other clinical parameters, such as the levels of C3/C4 and anti-dsDNA (data not shown).

**Figure 3:**
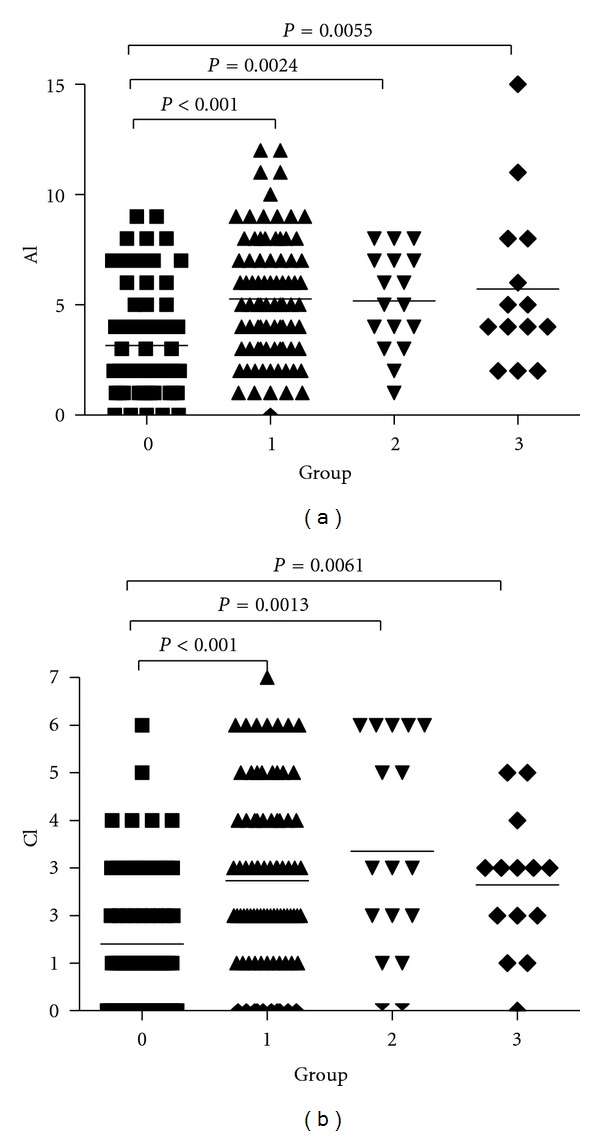
Lupus nephritis activity index (AI) and chronicity index (CI) in the four different B-cell groups of LN patients.

In the four different groups of LN patients, the activity and chronicity indices of grade 0 were significantly lower than the other grades (all *P* < 0.05) although there were no significant differences between grades 1, 2, and 3 (all *P* > 0.05) (Figure 3). No significant differences in immune complex deposition were identified in the four different groups of LN patients (all *P* > 0.05, data not shown).

## 4. Discussion

Lupus nephritis is one of the most frequent and serious complications in SLE patients. Although the pathogenesis of lupus nephritis is not clear, production of pathogenic antibodies is traditionally viewed as the principle contribution of B cells to the pathogenesis of immune-mediated glomerulonephritis [9, 16]. However, it is increasingly apparent that B cells have a much broader role in such diseases, functioning as antigen-presenting cells, regulators of T cells, macrophages, and dendritic cells and involved in the formation of local lymphocytic expansion [17]. 

The human B lymphocyte-specific marker, CD20, is a cell surface molecule which is widely expressed in B-cell differentiation subsets, including mature B cells and all subgroups of pre-B cells [18]. Steinmetz et al. observed that most locally infiltrating B cells in nephritis displayed a mature non-antibody-producing phenotype with antigen presenting capacity [10]. However, the report lacked a detailed description of the biopsy materials investigated. In this study, infiltrates were characterized by immunostaining using the B-cell marker CD20, the T-cell marker CD3, and CD21 as a marker of fDCs. Renal biopsies from 192 LN patients were analyzed and classified into four different groups according to their microanatomical structures. It was observed that B cells are predominantly detected in the tubulointerstitial compartment, with very few in the glomerular tuft. In previous studies of the characterization of the leukocyte subsets in human glomerulonephritis, such as IgA nephropathy and renal allograft rejection [19], B cells were rarely seen within glomeruli which is consistent with the results of this study. Although the number of B cells was found to be relatively low compared with T cells, there was a significant correlation between B cells and the degree of renal function. In this study, it was observed that SLEDAI, blood urea nitrogen, and serum creatinine levels were all significantly greater in the LN-B-cell group compared with the LN-non-B-cell group. Furthermore, intrarenal B cells were more likely to be associated with class IV LN and the activity and chronicity indices were also significantly higher in the LN-B-cell group compared with the LN-non-B-cell group. Therefore, it can be speculated that the formation of interstitial B-cell aggregates is a common response in LN and plays a pivotal role in renal injury. 

B cells are recruited to most chronically inflamed tissues and areas resembling secondary lymphoid tissue have been found in several forms of inflammatory kidney disease, including renal transplant rejection and glomerulonephritis [20]. However, the pathophysiological significance of these B cells remains to be clearly defined. Recent studies on lupus nephritis patients revealed that intrarenal B cells form local lymphoid tissue, described as tertiary lymphoid neogenesis, displaying a mature non-antibody-producing phenotype with antigen presenting capacity [10]. It is hypothesized that local proliferation in these germinal centers leads to the expansion and perpetuation of inflammatory cell aggregates resulting in persistence and chronicity of the renal inflammation. It has recently been reported that some lupus renal biopsies contain apparently functional germinal center- (GC-) like structures associated with *in situ* B-cell clonal expansion and somatic hypermutation [21]. However, in this study, intrarenal ectopic lymphoid tissue was not identified in any LN patients. These anomalies may result from differences in study design and ethnicity of the patient population. In accordance with previous studies, detailed classification of intrarenal lymphoid clusters was applied. However, no correlation was identified between clinical parameters and the presence of the three different classes of intrarenal B lymphoid aggregates. 

In renal allografts, the accumulation of CD20-positive B cells in the tubulointerstitium has been associated with steroid-resistant acute allograft rejection and with graft loss [18]. Rituximab is a CD20-specific chimeric antibody that depletes B cells, thereby inhibiting differentiation into antibody-producing plasma cells [22]. Depletion of local B cells inside the kidney could also prevent formation of intrarenal ectopic lymphoid structures. Improvement in disease activity in response to rituximab has been shown in some patients, although responses in many patients are only partial or completely fail [23–25]. These differences may reflect heterogeneity in LN pathogenesis and also emphasize the need for further investigation of the role of intrarenal B cells in LN. 

In order to further elucidate the mechanisms responsible for intrarenal B-cell formation, studies evaluating the role of chemokines such as BCA-1/CXCL13 and its specific receptor CXCR5, in the induction and maintenance of the spatial organization of B-cell infiltrates in LN biopsies have been performed [21, 26]. Local expression patterns of B-cell survival factors such as APRIL and BLyS are important factors for intrarenal B cells in lupus nephritis patients [26]. Studies indicate that antagonists of these chemokines, such as anti-BAFF/BCA-1 antibodies or corresponding receptors, may have therapeutic efficacy in patients with autoimmune disorders of this type [27]. It is hoped that an understanding of the expression patterns of renal B-cell and effectors would be beneficial to physicians in the choice of appropriate targeting reagents for different patients populations. It is speculated that intrarenal B cells form part of a local system that enhances the immunological response by functioning as antigen presenting cells, and as a source of cytokines promoting T cell proliferation and lymphatic neoangiogenesis. In this way, intrarenal B cells could enhance the local immune response to persisting autoantigens in the tubulointerstitium, resulting in persistence and chronicity of renal inflammation. 

## 5. Conclusions

This study revealed a correlation between clinical parameters and the presence of the four different intrarenal lymphoid aggregates in a large number of LN patients. It is hypothesized that intrarenal B cells enhance immunological responses and exaggerate the local immune response to persisting autoimmune damage in the tubulointerstitium. Investigation of follow-up biopsies from these patients is required to clarify this association and the function of intrarenal B cells in LN.

## Figures and Tables

**Table 1:** Demographics, clinical characteristics, and laboratory analysis of LN patients.

	LN-B-cell	LN-non-B-cell	*P* value
	*n* = 118	*n* = 74	
Sex (male/female)	17/101	8/66	0.513
Age (years)	34 ± 13	33 ± 13	0.526
SLE duration (months)	57 ± 60	43 ± 63	0.294
LN duration (months)	25 ± 37	26 ± 49	0.852
SLEDAI	14 ± 6	13 ± 5	0.042
Proteinuria (g/24 h)	3.13 ± 2.97	2.69 ± 2.56	0.311
Blood urea nitrogen (mmol/L)	10.43 ± 7.67	6.24 ± 3.40	0.000
Serum creatinine (*μ*mol/L)	89.91 ± 66.89	59.75 ± 22.05	0.000
Serum C3 (g/L)	0.51 ± 0.21	0.54 ± 0.26	0.427
Serum C4 (g/L)	0.082 ± 0.049	0.084 ± 0.076	0.827
Anti-dsDNA (positive/negative)^†^	72/11	44/9	0.506

^†^Anti-dsDNA antibodies were not detected in 35 patients in the LN-B-cell group and 21 patients in LN-non-B-cell group.

SLEDAI: systemic lupus erythematosus disease activity index; SLE: systemic lupus erythematosus; LN: lupus nephritis; anti-dsDNA: anti-double-stranded DNA antibody.

**Table 2:** Comparison of histologic parameters of LN-B-cell and LN-non-B-cell groups.

	LN-B-cell	LN-non-B-cell	*P* value
	*n* = 118	*n* = 74	
ISN/RPS classification			<0.001^∗^
I	1 (0.8)	6 (8.1)	
II	1 (0.8)	4 (5.4)	
III	5 (4.2)	8 (10.8)	
IV	56 (47.5)	15 (20.3)	
V	20 (16.9)	23 (31.1)	
III + V	15 (12.7)	13 (17.6)	
IV + V	19 (16.1)	5 (6.7)	
Activity index	5 (3–7)	2 (1–4.5)	0.000
Chronicity index	3 (1–4)	1 (0–2.5)	0.000

*P value for the difference in the ISN/RPS classification distribution between the two groups.
